# Making sense in biology: an appreciation of Julian Lewis

**DOI:** 10.1186/s12915-014-0057-5

**Published:** 2014-08-02

**Authors:** Arthur D Lander

**Affiliations:** Center for Complex Biological Systems, 2638 Biological Sciences III, University of California Irvine, Irvine, CA 92697-2300 USA

In every scientific field, there comes a point when progress stops being limited by the pace at which data can be collected, and instead becomes limited by the pace at which data can be understood. The experience can be jarring, as biologists have recently come to learn. Having benefited from extremely rapid advances in data gathering, they are now facing a bewildering influx of missionaries from computer science, physics, mathematics and engineering, who preach ‘big data science’, ‘machine learning’, ‘network science’, ‘reverse engineering’, ‘informatics’, ‘emergent behaviors’, and ‘design principles’ as the secrets to making sense of the mountains of data that are piling up daily.

**ᅟ:**
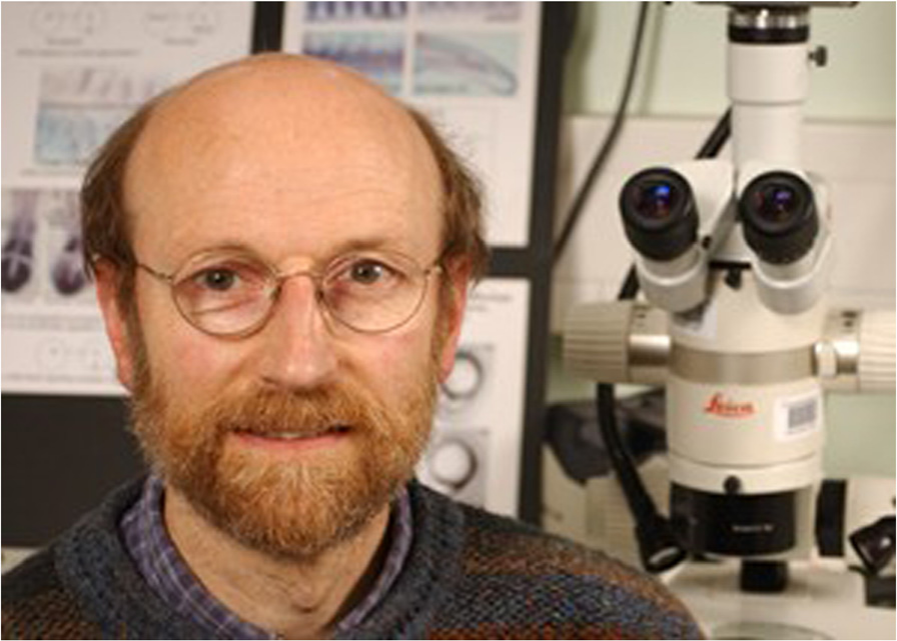
**Julian Lewis**

Standing in the midst of this intellectual bazaar, one might get the impression that biology has no traditions of its own for dealing with the complex or massive. In reality, biology has a rich history in this department, supported by the efforts of a handful of individuals who built the quantitative models that are the foundations upon which our most solid qualitative ideas rest. One of the most talented and effective of these individuals was Julian Lewis, who died at the end of April.

To many biologists (and an even greater number of students of biology and medicine), Julian’s impact was most directly felt through his contributions to *Molecular Biology of the Cell*, the ground-breaking textbook by Alberts *et al*. [1]. Initially brought in to contribute a single chapter on development to a first edition already underway, he so impressed the more senior authors with the clarity of his writing and his thought that he became a critical contributor to each of the book’s five editions over the last 30 years [2].

That clarity is very much in evidence in the research articles and reviews he published over a four-decade-long career. His work dealt with mechanism and theory of pattern formation, and included seminal contributions to the fields of limb development, Notch signaling, ear development, and somitogenesis (vertebrate body segmentation). Yet from my perspective, the significance of Julian’s specific contribution to those fields is dwarfed by something he contributed to biology in general, which was to teach us how to understand through modeling.

By this I don’t mean he was the first to use mathematics, or perform simulations, or fit data to complex equations - there has long been plenty of that in biology. What he did was to show biologists how the mathematical expression of ideas gives one power: power to explore the realm of the possible; power to make connections between phenomena; power to identify truly discriminating experiments; power to understand deeply.

As a postdoctoral researcher in the 1970s, Julian took what was then a semi-quantitative theory (concerning the assignment of positional information by morphogen gradients), and used simple mathematics to show just how difficult it would have to be for cells to position patterning thresholds precisely - paving the way for empirical studies in other laboratories over the next 35 years [3]. Later, in the 1990s, he collaborated with mathematicians Nick Monk and Philip Maini to show just what sorts of patterns Notch and Delta signaling could and could not produce [4], simultaneously constraining a great number of empirical studies and establishing the Notch/Delta system as a hotbed of quantitative biological research.

Indeed, it was his quantitative understanding of the morphogenetic possibilities of Notch signaling that led him to produce, in 2003, what many regard as his best modeling paper, and what I consider one of the best biological modeling papers of all time [5]. In this single-author work, Julian begins with his and others’ empirical observations on the role of Notch in the biochemical clock that produces the oscillations in gene expression that divide vertebrate mesoderm into segments (somites). He then explores, mathematically, exactly what conditions must obtain for regular, adjustable, noise-tolerant oscillations to occur, and to do so in a way that could fit existing data. The clarity of exposition (and modeling) in this paper set a standard for all future studies in somitogenesis and is, in my opinion, one of the reasons why experimental work in this rather complex area has progressed so rapidly.

Some of that experimental progress came, of course, from the Lewis lab itself, which put many quantitative predictions that came from his modeling to the test (see, for example, [6]). But I hesitate to leave the impression that modeling for him was merely a stepping stone to the next experiment. Although no one believed more strongly than he that a worthwhile model has to make testable predictions, his work showed us how modeling itself creates and constrains understanding. Without a model, a pile of data is simply a pile of data.

Seven years ago, I spent about nine weeks in the Lewis lab, on a ‘mini-sabbatical’ visit on which I had brashly invited myself following several enjoyable interactions with Julian at conferences. He generously offered me a spot in his office (really just a small corner of the lab behind a glass wall), and there we had many fine conversations, on topics ranging from Notch signaling to systems biology, and even cancer chemotherapy, which was by then a part of his daily routine. I was struck by his unusual combination of personality traits. On the one hand, he had an exceedingly gentle demeanor, treating students, postdocs and colleagues with uncommon kindness. On the other hand he had no patience at all for shoddy thinking or mediocre science, and could cut down a poorly conceived argument in a heartbeat.

At first I thought these two qualities to be at odds with each other, but I later realized that they were both part of what made him such a good scientist and modeler. The highest goal of modeling is to be able to articulate all that is entailed by everything that is possible, and Julian’s gentleness afforded him the openness of mind to consider and appreciate even the most far-fetched ideas. Yet, in the end, a map of the possible is only useful if it includes somewhere on it a bright red star with a sign saying, ‘You are (probably) here.’ It was Julian’s stringent standards of scientific rigor - and his deep knowledge of biology - that made it possible for him, repeatedly, to find that very spot.

I have known many modelers whose flights of fancy have produced works of great mathematical beauty but questionable biological relevance. I have also known many quantitative biologists who, through dogged adherence only to what can be inferred from the data at hand, lacked the imagination to conceive of what ‘must’ or ‘should’ be, before knowing it is actually there. Julian Lewis was the only scientist I have known whose combination of imagination and discipline allowed him consistently to avoid both these pitfalls.

This year *BMC Biology* is launching a new series on modeling in biology, following on a beautifully written inaugural review from Jeremy Gunawardena [7], in which Julian’s work on the somite oscillator is among several featured stories. That a general biology journal would run a series on modeling is in many ways a tribute to Julian’s singular impact. As biology gropes its way through a world in which making sense, as opposed to making discoveries, takes an ever-increasing amount of our attention, we should be grateful to him for having helped illuminate the path.

## References

[1] Alberts B, Johnson A, Lewis J, Raff M, Roberts K, Walter P (2008). Molecular Biology of the Cell.

[2] Serpente N (2013). Beyond a pedagogical tool: 30 years of Molecular Biology of the Cell. Nat Rev Mol Cell Biol.

[3] Lewis J, Slack JM, Wolpert L (1977). Thresholds in development. J Theor Biol.

[4] Collier JR, Monk NA, Maini PK, Lewis JH (1996). Pattern formation by lateral inhibition with feedback: a mathematical model of delta-notch intercellular signalling. J Theor Biol.

[5] Lewis J (2003). Autoinhibition with transcriptional delay: a simple mechanism for the zebrafish somitogenesis oscillator. Curr Biol.

[6] Giudicelli F, Ozbudak EM, Wright GJ, Lewis J (2007). Setting the tempo in development: an investigation of the zebrafish somite clock mechanism. PLoS Biol.

[7] Gunawardena J (2014). Models in biology: ‘accurate descriptions of our pathetic thinking’. BMC Biol.

